# Single-molecule photobleaching reveals increased MET receptor dimerization upon ligand binding in intact cells

**DOI:** 10.1186/2046-1682-6-6

**Published:** 2013-06-03

**Authors:** Marina S Dietz, Daniel Haße, Davide M Ferraris, Antonia Göhler, Hartmut H Niemann, Mike Heilemann

**Affiliations:** 1 Institute of Physical and Theoretical Chemistry, Johann Wolfgang Goethe-University, Max-von-Laue-Str. 7, 60438 Frankfurt, Germany; 2 Structural Biochemistry, Department of Chemistry, Bielefeld University, Universitaetsstr. 25, 33615 Bielefeld, Germany; 3 Department of Pharmaceutical Science, University of Eastern Piedmont “Amedeo Avogadro”, Via Bovio 6, Novara, 28100, Italy; 4 Department of Biotechnology and Biophysics, Julius-Maximilians University, Am Hubland, Biozentrum, Wuerzburg, 97074, Germany

**Keywords:** MET receptor, Dimerization, Single-molecule photobleaching, Fluorescence correlation spectroscopy, Fluorescence, Signal transduction

## Abstract

**Background:**

The human receptor tyrosine kinase MET and its ligand hepatocyte growth factor/scatter factor are essential during embryonic development and play an important role during cancer metastasis and tissue regeneration. In addition, it was found that MET is also relevant for infectious diseases and is the target of different bacteria, amongst them *Listeria monocytogenes* that induces bacterial uptake through the surface protein internalin B. Binding of ligand to the MET receptor is proposed to lead to receptor dimerization. However, it is also discussed whether preformed MET dimers exist on the cell membrane.

**Results:**

To address these issues we used single-molecule fluorescence microscopy techniques. Our photobleaching experiments show that MET exists in dimers on the membrane of cells in the absence of ligand and that the proportion of MET dimers increases significantly upon ligand binding.

**Conclusions:**

Our results indicate that partially preformed MET dimers may play a role in ligand binding or MET signaling. The addition of the bacterial ligand internalin B leads to an increase of MET dimers which is in agreement with the model of ligand-induced dimerization of receptor tyrosine kinases.

## Background

The MET receptor tyrosine kinase (RTK) and its physiological ligand hepatocyte growth factor/scatter factor (HGF/SF) play an essential role in vertebrate development as well as in tissue regeneration including proliferation, migration, cell survival and differentiation [1]. Deregulated MET activation is found in different forms of cancer. In addition, pathogenic bacteria exploit MET and its signaling pathways for infection. *Listeria monocytogenes*, the causative agent of human listeriosis, initiates its entry into normally non-phagocytic host cells with the surface protein internalin B (InlB) [2,3]. InlB is non-covalently attached to the bacterial cell wall but also released into the medium [4]. Soluble InlB binds and activates MET leading to similar cell responses as those elicited by HGF/SF [3,5].

Ligand-induced dimerization of the receptor ectodomain constitutes the widely accepted paradigm for the activation of RTKs [6]. However, there are exceptions like the insulin receptor, which is constitutively dimeric. The structural basis and the molecular mechanism of receptor activation vary widely between different RTKs and need to be worked out experimentally for each pair of receptor and ligand [6,7]. In the case of the MET receptor and its physiological ligand HGF/SF, the mechanism of receptor activation is still not completely understood. Structural studies have not yet provided a clear-cut picture of the signaling-active complex [8] and small angle X-ray scattering showed that active HGF/SF only forms a 1:1 complex with the complete MET ectodomain in solution [9]. There is ample evidence showing that forced MET dimerization, e.g. by antibodies or other dimeric ligands, can activate the receptor (reviewed in [7]). However, even in the absence of ligand, a substantial fraction of the endogenous MET receptors from various tumor cell lines can be cross-linked into dimers [10] and larger multimers were found on different cell lines [11]. Hence, it remains an open question whether MET activation by HGF/SF and InlB really proceeds through dimerization of initially monomeric receptors.

**Figure 1:**
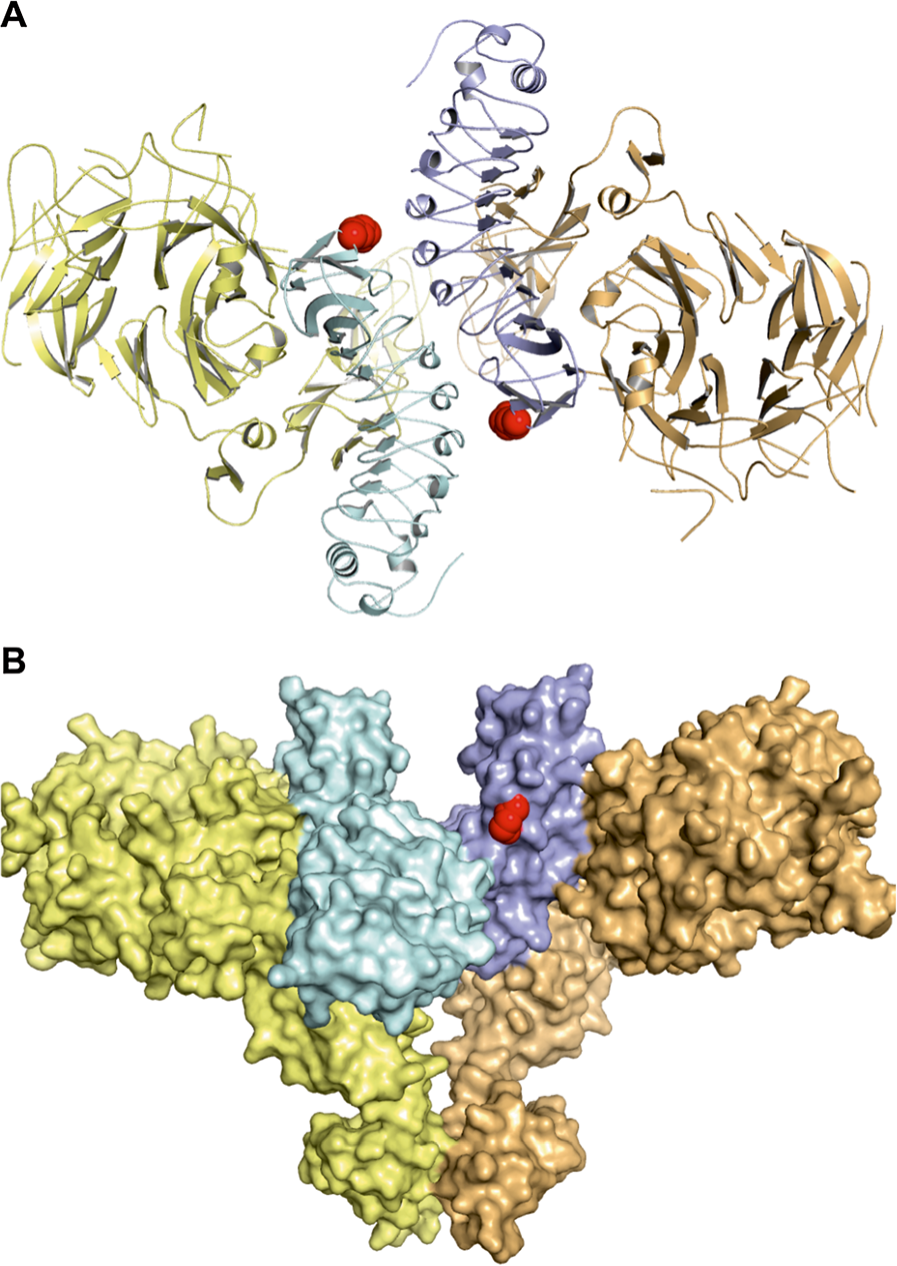
**Crystal structure of the MET-InlB complex (PDB ID 2UZY). **MET is shown in yellow and orange, InlB_321 _in cyan and blue. L280, which was mutated to cysteine for attachment of ATTO647N, is shown in red. (**A**) Top view along the 2-fold axis onto the plane of the membrane. (**B**) Side view with the membrane-proximal end of MET pointing down.

Like HGF/SF, InlB is a multi-domain protein. The high-affinity binding site for MET is located within the N-terminal internalin domain of InlB (amino acids 36–321; InlB_321_) [3,5]. The soluble, monomeric internalin domain of InlB is sufficient to induce MET phosphorylation in various cell lines [5,12,13]. In analogy to other RTKs, this receptor activation is expected to proceed through ligand-induced dimerization of the MET extracellular domain. Crystallography revealed a biologically plausible 2:2 complex of InlB_321_ bound to a large part of the MET ectodomain (Figure 1) and mutagenesis and cellular assays established that a contact stabilizing this 2:2 complex is important for InlB-mediated MET activation [5,13], lending further support to the proposed MET dimerization upon binding of InlB [7]. In solution, however, the complex between InlB_321_ and the complete MET ectodomain is strictly monomeric and 2:2 complexes were not observed by a variety of biochemical and biophysical methods [5,14]. The question, whether binding of InlB_321_ to MET on cells does actually induce receptor dimerization has not yet been addressed experimentally.

Studying receptor oligomerization in a native cellular environment is challenging. Among the different approaches, single-molecule fluorescence microscopy is an ideal tool as it can resolve heterogeneities and subpopulations [15]. For example, receptor oligomerization was studied using single-molecule intensity analysis in living cells [16]. Single-molecule photobleaching has developed to a reliable technique to investigate the number of subunits in protein complexes and determine the stoichiometry of membrane receptors [17-20].

So far, fluorescence microscopy has been largely neglected in the study of MET and HGF/SF. Two fusions of a fluorescent protein to the C-terminus of MET were reported [21,22], but the recombinant proteins were overexpressed from strong promoters which limits their utility for studying ligand-induced MET dimerization, as strong overexpression is known to result in ligand-independent MET dimerization [10,23,24]. Accordingly, these fluorescent MET fusions have not been used for mechanistic analysis at the molecular level. Likewise, site-specific fluorescent labeling of HGF/SF has, to the best of our knowledge, not been reported so far.

Here, we use single-molecule photobleaching to study the oligomerization of endogenous MET on intact HeLa cells by targeting with a fluorophore-labeled ligand, InlB_321_. We find that MET exists partially as monomer prior to stimulation, and observe an increased amount of dimers upon stimulation by InlB_321_. Our results contribute to the current understanding of the mechanism of signal transduction by the MET receptor.

## Results and discussion

### Receptor density determined by single-molecule super-resolution imaging

Single-molecule photobleaching experiments require a molecular density sufficiently low to ensure the detection of single, spatially separated receptor clusters. Often, the spatial density of an endogeneous membrane receptor is too high. For example, the number of MET receptors per cell reported varies widely from hundreds to a hundred thousand per cell [25-29]. We determined the total number of endogenous MET receptors on HeLa cells using direct stochastic optical reconstruction microscopy (*d*STORM) [30]. This technique provides a sufficient spatial resolution in order to resolve individual receptor sites and thus to estimate the total number of receptors per cell. We used a polyclonal antibody directed against the human MET ectodomain and a secondary antibody labeled with the photoswitchable fluorophore Alexa Fluor 647, and quantified the number of MET receptors per cell (Additional file 1: Figure S1). We found an average receptor density of 6.5 ± 0.6 (s.d.) molecules / μm^2^, roughly corresponding to 4600 to 8700 MET molecules per HeLa cell with an estimated surface ranging from 700 to 1300 μm^2^. This value is within previously published numbers of receptors [25-29]. However, the receptor density is too high for stoichiometric labeling of MET for single-molecule photobleaching, as the fluorescence signal of single receptors would overlap. We thus used a fluorophore-labeled ligand, InlB_321_, at concentrations which only yield a fractional labeling of MET.

### Site-specifically, fluorescently labeled InlB_321_ binds MET with nanomolar affinity ***in vitro***

**Figure 2:**
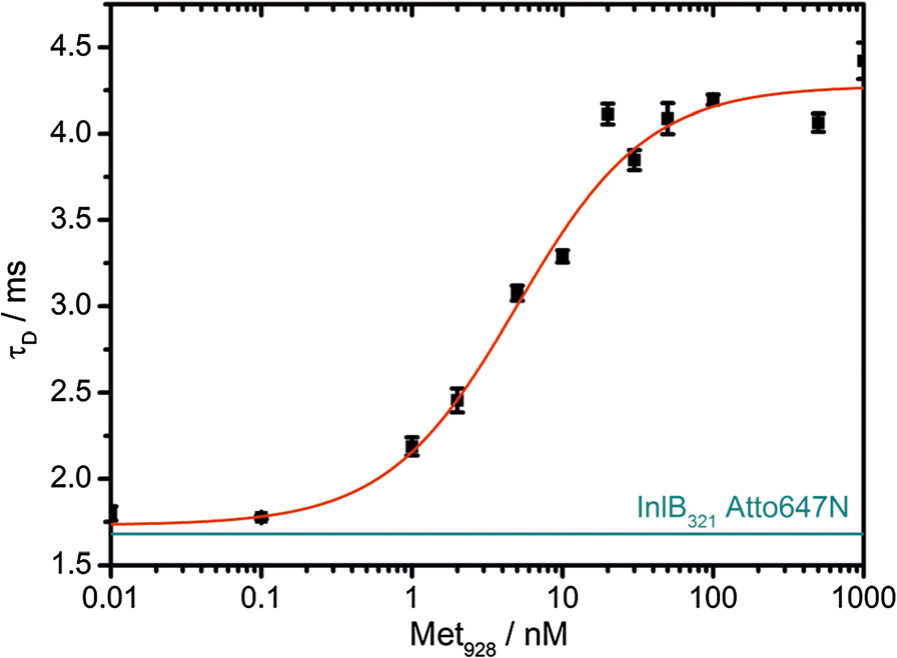
**FCS binding curve of MET**_**928 **_**titrated against InlB-ATTO647N. **The diffusion time τ_D _of the complex is plotted against the receptor concentration. The binding curves are fitted by a Langmuir 1:1 binding model. The diffusion time of InlB-ATTO647N in the absence of MET is marked as green line. The inflection point represents the dissociation constant K_d_ = 5.0 ± 0.8 nM.

For single-site labeling of InlB_321_ we mutated L280 to cysteine, because its side chain is surface exposed and it is not involved in any binding interface of the 2:2 InlB:MET complex (Figure 1). ATTO647N, which we used due to its excellent photophysical properties, was attached to C280 using maleimide coupling resulting in singly labeled InlB_321_. The degree of labeling (DOL) was determined from absorption measurements to be 0.5. We will henceforth refer to this protein as InlB-ATTO647N. We used fluorescence correlation spectroscopy (FCS) to assess whether InlB-ATTO647N is still able to interact with the MET receptor. The MET ectodomain (MET_928_) was titrated in a concentration range from 0.01 nM to 1–2 μM against 1 nM InlB-ATTO647N. After a minimum of 3 h incubation, FCS curves were recorded and approximated with a two-dimensional diffusion model [31]. The resulting diffusion time plotted against the concentration of MET_928_ was fitted to a 1:1 binding model resulting in a dissociation constant K_d_ = 5.0 ± 0.8 nM (Figure 2). Thus, our FCS studies revealed that InlB-ATTO647N is functional *in vitro.* We had previously attempted to determine the K_d_ for InlB_321_ and MET using surface plasmon resonance (SPR) with either InlB coupled to the sensor chip and MET as analyte [32] or MET coupled to the chip and InlB as analyte [33] yielding values of 20–30 nM and ~150 nM, respectively. Enzyme linked immune sorbent assays with immobilized MET_928_ and dimeric GST-InlB_321_ fusion proteins [33,34] or monomeric InlB_321_[32] yielded half-maximal binding roughly between 1 nM and 5 nM. In these experiments artifacts might arise from immobilization (e.g. causing steric hindrance preventing conformational changes), from the use of the dimeric GST-InlB (reflecting avidity rather than true affinity) or from effects like mass transport in SPR. We assume that the K_d_ reported here represents the most reliable value, as the FCS measurements were carried out in solution and the fluorescent label is not expected to interfere with MET binding (Figure 1).

### Visualization of single MET receptors

We used InlB-ATTO647N in order to study the multimerization state of the MET receptor in HeLa cells before and after stimulation. The resting state was imaged by chemically fixing serum-starved HeLa cells before incubation with InlB-ATTO647N. This approach is based on the assumption that InlB-ATTO647N cannot induce receptor dimerization if added to the cells only after fixation. InlB-stimulated cells were prepared by incubating HeLa cells with InlB-ATTO647N at 4°C for 1 h prior to chemical fixation. The fluorescence signal was recorded at the basal membrane of the cell in total internal reflection (TIR) mode. Under our experimental conditions of 10 nM concentration of InlB-ATTO647N, we found a receptor density of 0.6 ± 0.2 (s.d.) molecules / μm^2^. Hence, only about 10% of all endogenous receptors were labeled with InlB-ATTO647N, due to a partial occupation of the receptor with ligand (expected to be 67% at a ligand concentration of 10 nM assuming a *K*_d_ of 5 nM), the DOL of InlB-ATTO647N (0.5) and additional factors such as ligand adsorption to the glass slide or photobleaching of the fluorophore prior to the experiment. In addition, we did observe few larger clusters which were excluded from single-molecule analysis.

### Single-molecule photobleaching data indicate receptor dimerization

**Figure 3:**
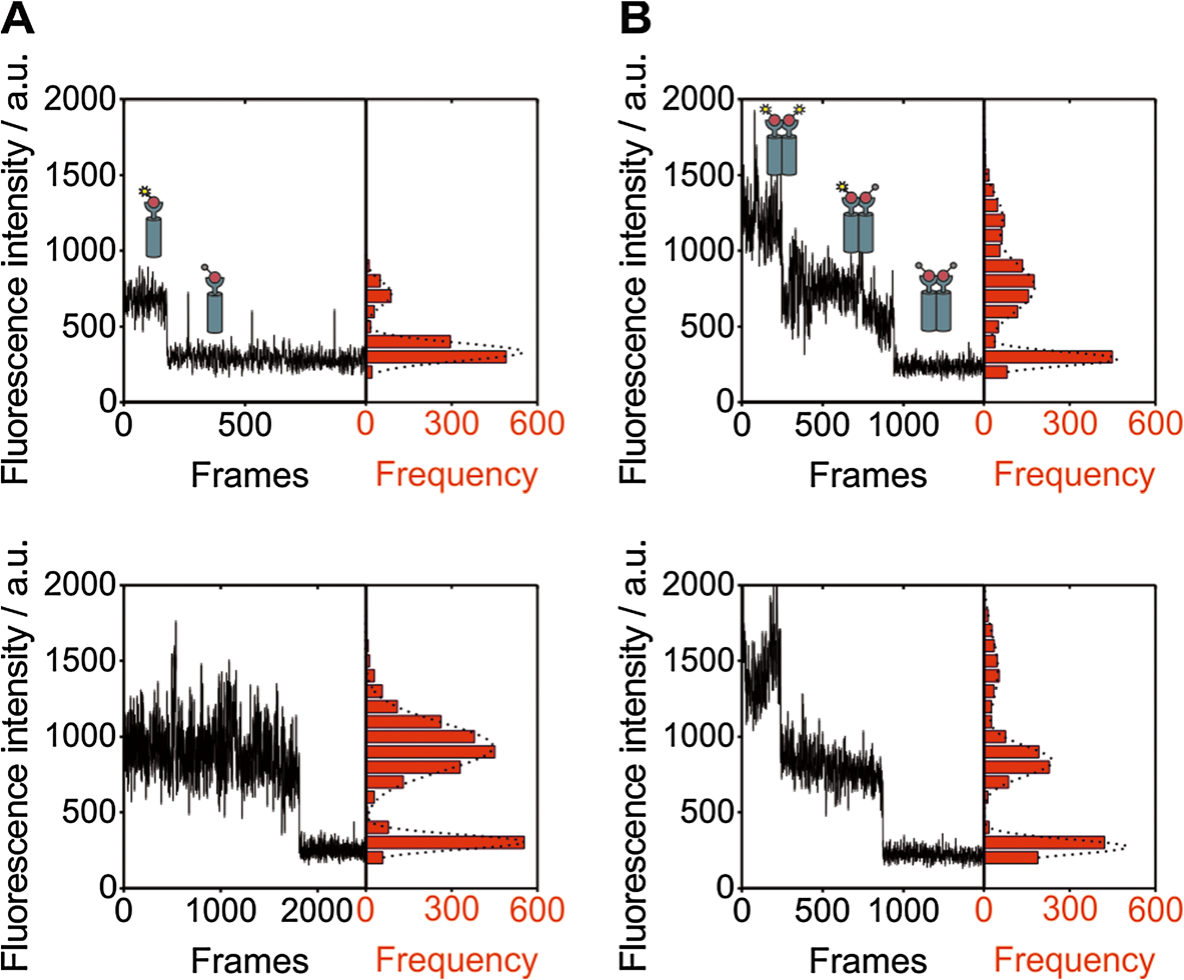
**Single-molecule photobleaching of MET receptor. **(**A**) Fluorescence intensity trajectories found for InlB-ATTO647N in uninduced HeLa cells. In most cases, one-step photobleaching was observed. The histograms on the right show the distribution of fluorescence intensities. (**B**) InlB induced cells show an increase in two-step photobleaching of fluorescence spots, reporting on MET dimers.

We observed the step-wise photobleaching by analyzing fluorescence intensity over time in single fluorescent spots (Figure 3). In the case of cells that were fixed prior to addition of InlB_321_, we mainly observed one-step photobleaching (Figure 3A). In the case of InlB_321_ stimulation before cell fixation, two-step photobleaching indicated the presence of two InlB in a diffraction-limited spot and thus receptor dimerization (Figure 3B).

**Figure 4:**
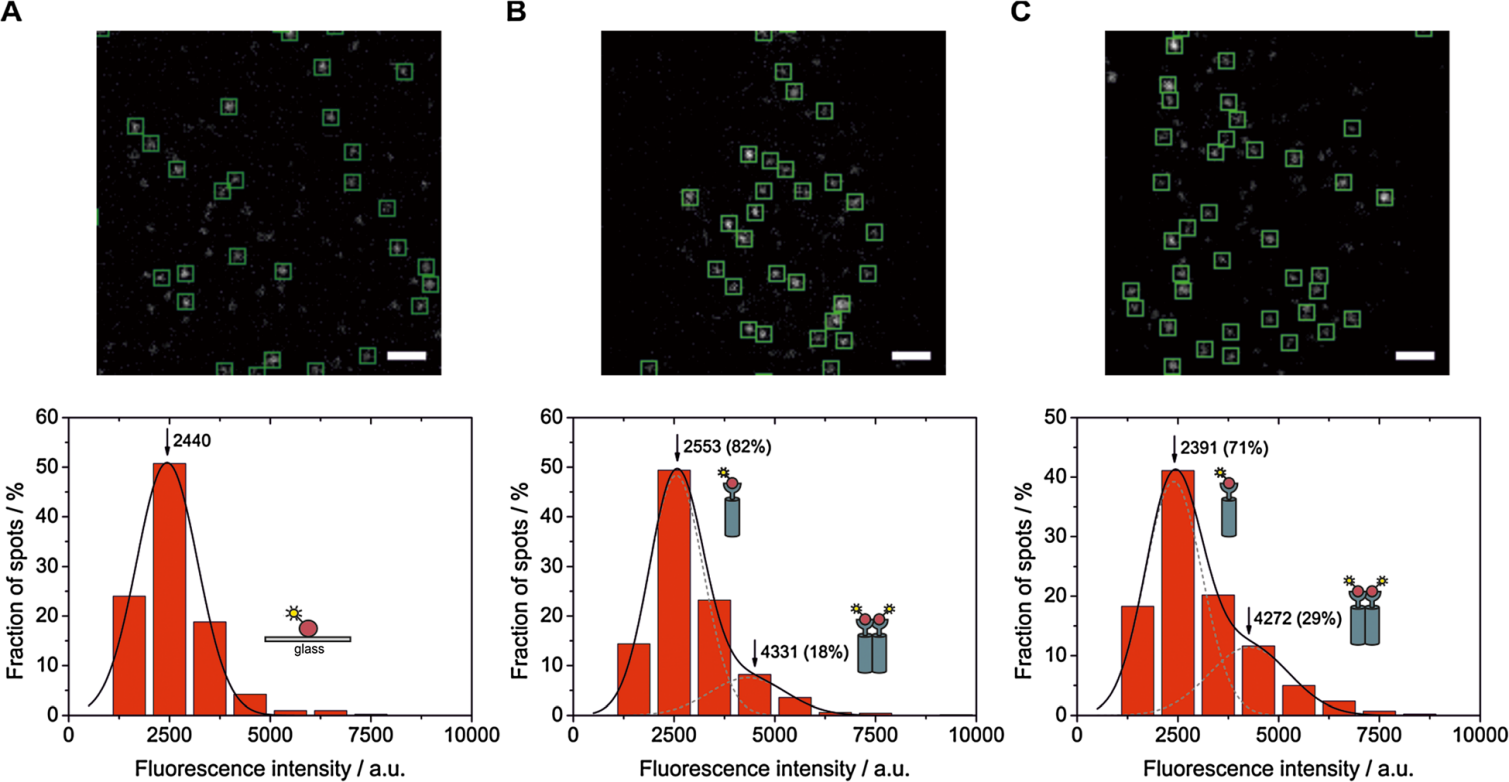
**Binding of InlB to MET induces receptor dimerization. **Single-molecule data were background-corrected and single-fluorophore intensity distributions extracted. (**A**) Individual fluorescence spots of single InlB-ATTO647N adsorbed to an L-lysine coated glass surface (top). Fluorescence spots selected for intensity analysis are marked with green squares (7x7 pixels) (scale bar 2 μm). The intensity distribution of individual InlB-ATTO647N molecules (n = 404) was well fitted with a single Gaussian function. (**B**) TIRF image of an uninduced cell. The distribution of fluorescence intensities was fitted by two Gaussian functions (n = 527 spots from 17 cells were analyzed). (**C**) TIRF image of an induced cell. The intensity distribution shows an increase of the fraction of receptor dimers (n = 421 spots from 10 cells). Numbers on histograms indicate maxima and fractions.

We evaluated the single-molecule data quantitatively by analyzing the fluorescence intensity in single spots using a similar approach as published earlier [19]. As a control, we analyzed a single-molecule surface of InlB-ATTO647N on an L-lysine coated glass surface. The TIRF image showed clearly separated fluorescence spots, and the single-molecule intensity distribution can be fitted with a single Gaussian function (Figure 4A). In the case of uninduced cells, this distribution can be approximated with two Gaussian functions where the standard deviation σ_2_ of the second Gaussian is $\sqrt{2}\sigma_{1}$ with σ_1_ being the standard deviation of the monomeric intensity distribution (Figure 4B). We observed 82% spots originating from monomeric receptors and 18% dimers. In the case of InlB-induced cells, this distribution changed in favor of the dimeric fraction which increased to 29% (Figure 4C). These results suggest that MET is present both as monomers and as dimers in the absence of InlB-ATTO647N, and that binding of InlB-ATTO647N increases the fraction of dimers significantly.

The actual fraction of dimers is clearly underestimated in the single-molecule intensity analysis due to non-stoichiometric labeling of MET, although this cannot be easily calculated from the current data. Notwithstanding this uncertainty, single-molecule microscopy clearly showed that InlB stimulation results in increased MET dimerization on intact cells. This resolves the apparent contradiction that a MET ligand induces receptor phosphorylation in cellular assays although it does not promote dimerization of the MET ectodomain in solution. Membrane anchorage of MET in cellular assays reduces the dimensionality of diffusion from 3 dimensions in solution to 2 dimensions in the plane of the membrane. Reducing the dimensionality of diffusion is an important concept in many biological processes that can reduce diffusion times [35] or increase apparent affinities [36]. We can thus envisage the following order of events: monomeric InlB binds with high affinity to MET receptors, which are present as both monomers and dimers on the cell. The 1:1 complexes diffuse two-dimensionally within the membrane plane and eventually form low-affinity 2:2 complexes [37].

Our observation by fluorescence microscopy of MET dimers on cells that were not treated with HGF/SF or InlB is consistent with previously reported data from chemical cross-linking. In several tumor cell lines, endogenous MET can be cross-linked into dimers in the absence of ligand [10,38]. An important role of the Sema domain for ligand-independent MET dimerization was shown [10], and Sema domains from semaphorins generally form homodimers [39]. However, there is no crystallographic evidence for homodimerization of the MET Sema domain so far. Instead, one could also envisage a mechanism of ligand-independent MET dimerization that does not involve the MET ectodomain. A MET construct lacking the complete ectodomain is constitutively active, suggesting that the transmembrane domain and/or the juxtamembrane and kinase domain of MET may promote dimerization [40]. Both the transmembrane and the intracellular domain also contribute to dimerization of other RTKs like EGFR [41,42].

This leaves us with several questions: What is the importance of preformed MET dimers? Do ligands preferentially bind to preformed receptor dimers? Are the preformed dimers signaling active or do they require ligand binding for signaling? Right now we can only speculate about these issues for MET. For other receptor systems, however, answers are already available. For some cytokine receptors like the erythropoietin receptor or the growth hormone receptor dimerization is required but not sufficient for signaling. In addition, a particular orientation of the two receptors in the dimer seems to be important. These receptors appear to exist as preformed dimers and ligand binding may result in a rotational or translational movement of the protomers relative to each other [43,44]. Very recently, a computational study reported such relative movements of the receptor molecules as a model for cytokine receptor activation [45]. Single molecule fluorescence microscopy has also provided evidence for preformed dimers of unliganded EGFR on cells, but ligand binding was still required for signaling [46,47].

## Conclusion

Our data clearly show that the receptor tyrosine kinase MET exists as both monomers and preformed dimers in the absence of ligand. To our knowledge we have experimentally shown for the first time that the addition of the bacterial ligand InlB leads to increased MET dimerization. This result is consistent with the well-established model of ligand-induced dimerization of receptor tyrosine kinases. The importance of the preformed dimers remains elusive. Clarifying the complete mechanism of MET receptor activation will require a multidisciplinary approach employing complimentary experimental techniques. For sure, single-molecule fluorescence microscopy has the potential to contribute exciting insights to this endeavor.

## Methods

### Expression and purification of internalin B

InlB_321_ was expressed as tobacco etch virus (TEV) protease cleavable glutathione-S-transferase (GST) fusion protein. In addition to the K280C mutation, the naturally occurring C242 was mutated into alanine in order to prevent the formation of undesired intramolecular disulfide bonds during folding. Banerjee et al. [12] had already shown that this mutation does not impair MET binding. The C242A and K280C mutations were introduced into the previously described pETM30 vector [5] using QuikChange^®^ mutagenesis kit (Stratagene). Transformed *Escherichia coli* BL21-CodonPlus(DE3)-RIL were grown in lysogeny broth (LB) medium containing kanamycin and chloramphenicol at 37°C to an optical density at 600 nm (OD_600_) of 0.6. After induction with 0.1 mM isopropyl β-D-1-thiogalactopyranoside bacteria were incubated with shaking at 20°C overnight. Cells were harvested by centrifugation. The bacterial pellet was washed with phosphate buffered saline (PBS), resuspended in PBS containing a complete protease inhibitor cocktail tablet (Roche) and DNaseI (2.4 μg/mL), and lysed by three passes through a French pressure cell press. After centrifugation, the cleared lysate was applied for 1 h at 4°C to 10 mL of glutathione sepharose affinity matrix (GE Healthcare) equilibrated in PBS. After extensive washing with first PBS and then TEV protease cleavage buffer (50 mM Tris, pH 8.0, 0.5 mM EDTA, 20 mM NaCl, 1 mM DTT), the resin was resuspended in 40 mL of TEV cleavage buffer and 40 μL of 1 M DTT were added. The target protein was cleaved off from the GST-tag by incubation with 0.5 mg of TEV protease at room temperature overnight. The liberated target protein was eluted and further purified by anion exchange chromatography. InlB_321_ was applied to Source Q 15 (GE Healthcare) equilibrated in 20 mM Tris, pH 7.5 and eluted with a linear salt gradient up to 300 mM NaCl over 10 column volumes.

### Labeling of internalin B with ATTO647N

For fluorescence labeling, fresh InlB_321_ was used directly after elution from the anion exchange column. The main fraction from Source Q 15 was covered with nitrogen and tris(2-carboxyethyl)phosphine (TCEP) was added to a final concentration of 0.5 mM. For each labeling reaction, 100 μg of ATTO647N maleimide (ATTO-TEC) dissolved in 5 μL of dry dimethylformamide (DMF) were used. Immediately before the labeling, a 3-fold molar excess of TCEP was added to the protein and a 3-fold molar excess of ATTO647N maleimide over protein was incubated with InlB_321_ in the dark at room temperature for 1 h. Excess fluorophore was removed using a PD10 desalting column (GE Healthcare) equilibrated in PBS. The elution of the PD10 column was fractionated. Coomassie stained sodium dodecyl sulfate polyacrylamide gel (SDS-PAGE) was used to assess protein purity and to roughly estimate the degree of labeling (DOL). The protein concentration and the DOL were determined spectrophotometrically using absorption at 280 and 644 nm. The DOL was calculated using the formula DOL = [A_644_*ϵ_280_(protein)] / ([A_280_ – A_644_*CF]*ϵ_644_(fluorophore)). The correction factor CF corrects for the absorption of ATTO647N at 280 nm and its value of 0.05 was experimentally determined by calculating A_280_/A_644_ measured for pure ATTO647N maleimide. Labeled InlB_321_ was stored at -20°C in the dark.

### Expression and purification of MET_928_

The soluble MET ectodomain (MET_928_) was expressed in glycosylation deficient Chinese hamster ovary (CHO) lec8 cells and purified essentially as described previously [5,9,48]. Size exclusion chromatography on a Superdex 200 column (GE Healthcare) equilibrated in PBS was used as final purification step in order to obtain only monomeric MET_928_ that was used for fluorescence correlation spectroscopy.

### Cell culture

HeLa cells were cultured on chamber slides and grown for 24 h in RPMI 1640 medium containing 100 IU/mL penicillin, 100 μg/mL streptomycin, 2 mM L-glutamine and 5% fetal calf serum at 37°C in 5% CO_2_. Before the experiment, the cells were serum starved for 24 h.

### Binding of InlB to MET on HeLa cells

For resting cells, HeLa cells were fixed with 4% formaldehyde in phosphate buffered saline (PBS) (pH 7.4) for 10 min. After washing with PBS, the cells were incubated with 10 nM of the InlB-ATTO647N construct in PBS for 1 h at 4°C. For InlB induced cells, living HeLa cells were cooled for 10 min at 4°C to prevent internalization of MET upon InlB binding. 10 nM InlB-ATTO647N in RPMI medium were added and binding occurred within 1 h at 4°C. Afterwards, the cells were washed with ice cold PBS and fixed with 4% formaldehyde for 10 min.

### Immunofluorescence

HeLa cells were grown and serum starved as described above. To prevent *de novo* protein synthesis, cells were incubated with 50 μM cycloheximide (Sigma-Aldrich) in starvation medium for 2 h. The cells were fixed with 4% formaldehyde for 10 min. After washing with PBS, cells were blocked with blocking buffer (5% BSA in PBS) for 1 h at room temperature. The polyclonal primary antibody directed against the complete ectodomain of human MET (AF276, R&D Systems) was added to the cells at a concentration of 2 μg/mL in blocking buffer. Cells were incubated for 2 h at room temperature and washed three times with PBS for 5 min. Secondary antibody labeled with Alexa Fluor 647 (rabbit anti-goat IgG, Invitrogen) was added at a concentration of 2 μg/mL in blocking buffer. After 1 h incubation, cells were washed three times with PBS.

### Fluorescence correlation spectroscopy

The FCS measurements were performed using a home-built confocal microscope (for detailed information see [31,49]). ATTO647N was excited at 638 nm using a diode laser (Cube 635, Coherent). Photobleaching was avoided by using very low excitation intensities (200 μW, measured at the back aperture of the objective). The laser beam was coupled into an oil-immersion objective (63×, NA 1.4; Zeiss) by a dichroic beam splitter (645DLRP, Omega Optics). The emission light was collected by the same objective, passed a band-pass filter (700DF75, Omega Optics), separated into two beams using a cubic non-polarizing beamsplitter (Linos) and coupled into two multi-mode optical fibers with a diameter of about 100 μm. The signal was detected by the active area of two single-photon avalanche photodiodes (AQR-14, Perkin Elmer) and the signals of these photodiodes were cross-correlated (5 min for each measurement) using a digital real-time multi-tau correlator device (ALV-6010, ALV GmbH) with a time resolution of 6.25 ns.

For sample preparation, the dye-labeled InlB_321_ was diluted to a final concentration of 1 nM in 0.05% Tween-20 in PBS. The MET_928_ ectodomain was added in different end concentrations in the range from 0.01 nM to 1–2 μM. The total volume in each reaction tube was 100 μL. After thoroughly mixing, the complex formation was allowed to occur for at least 3 h at room temperature. The reaction mixtures were transferred onto a microscope slide and covered with a coverslip. Each measurement was done at a constant depth of 40 μm from the glass surface. The temperature of the objective was kept constant at 20°C by a custom-made heating block.

FCS data analysis is described in detail elsewhere [31]. In brief, fluctuations in the fluorescence signal I(t) due to diffusion of the dye labeled MET_928_-InlB_321_ complex in and out of the detection volume were analyzed via the second-order autocorrelation function:

$$G\left(\tau \right)=\frac{I\left(t\right)I\left(t+\tau \right)}{I{\left(t\right)}^{2}}$$

Here, 〈 〉 denotes the time average over the total observation time. Approximation of this equation can be done by using a two-dimensional diffusion model for a single species in combination with a stretched exponential decay accounting for photophysical processes.

$$G\left(\tau \right)=\frac{1}{N}{\left(1+\frac{\tau }{\tau_{D}}\right)}^{-1}\left(1+K\cdot \text{exp}{\left(-\frac{\tau }{\tau_{\text{rel}}}\right)}^{\beta }\right)$$

with N the number of detected molecules, τ_D_ the diffusion time, K the amplitude, τ_rel_ the rate constant, and β the stretch parameter of a decay accounting for photophysical processes. The diffusion time τ_D_ depends on the dimensions of the detection focus ω_xy_ in x,y-dimension and the diffusion constant D as

$$\tau_{D}=\omega_{\mathrm{xy}}^{2}/4D$$

By measuring the diffusion times at different concentrations of MET_928_, a complete binding curve could be determined. Plotting the diffusion time τ_D_ against the MET_928_ concentration and fitting with a simple 1:1 binding model allows estimation of the dissociation constant K_d_ of the MET_928_-InlB_321_ complex.

$$\tau_{D}=\tau_{\text{InlB}}+\left(\tau_{\text{InlB}-\text{MET}}-\tau_{\text{InlB}}\right)\frac{\left[\text{MET}\right]}{K_{d}+\left[\text{MET}\right]}$$

where τ_InlB_ and τ_InlB - MET_ are the diffusion times of free InlB and of the MET_928_-InlB_321_ complex.

### Single-molecule microscopy

The experimental setup consisted of an inverted microscope (Olympus IX-71) equipped with an oil-immersion objective (60x, NA 1.45, Olympus). The 647 nm laser line from an argon krypton laser (Coherent) was selected by an acousto-optic tunable filter (AAOptics), passed a dichroic beamsplitter (FF560/659, Sembrock) and focused onto the back focal plane of the microscope lens. Total internal reflection fluorescence (TIRF) configuration allowed for near-surface illumination of ATTO647N or Alexa Fluor 647, respectively. The emission light is filtered in the detection path by a bandpass (ET700/75, AHF Analysentechnik) and a longpass filter (LP647RU, AHF Analysentechnik) and detected on an electron-multiplying charge-coupled device (EMCCD) camera (Ixon DU897, Andor).

### Single-molecule surface of InlB-ATTO647N

Glass coverslips were cleaned by incubating with 1 M HCl overnight. After washing thoroughly with bidistilled water and 100 mM NaHCO_3_ (pH 8.5), the surface was covered with 0.1% poly-L-lysine (Sigma-Aldrich) in water. The coverslips were incubated for 10 min at room temperature. The solution was removed and the poly-L-lysine coated glass surface was dried. The surface was washed with 100 mM phosphate buffer (pH 7.3) before incubation with InlB-ATTO647N (0.1 nM) in phosphate buffer for 5 min. Finally, the glass surface was washed with PBS.

### Single-molecule photobleaching

Single-molecule photobleaching was performed in TIR mode. For enhancing the photostability of ATTO647N 100 mM β-mercaptoethylamine (MEA) in PBS was added. Movies of 1000 to 3000 frames were recorded with 33 Hz using an irradiation intensity of about 0.3 kW/cm^2^.

To analyze photobleaching steps, movies were background-corrected with the rolling ball method using ImageJ (NIH). Photobleaching intensity time traces were extracted from regions of 7×7 pixel size. For the analysis of the fluorescence intensity distribution the open-source image analysis software ICY (Institut Pasteur) was used [50]. The background corrected first frame of each movie was analyzed using a custom script (Fabrice de Chaumont) which includes the Spot Detector plugin in ICY. A threshold was set, a 7×7 pixels region of interest was built around the center of mass of each detection and the overall intensity was calculated. Frequency distributions of the fluorescence intensities were generated with OriginPro 8.6G (OriginLab).

### Receptor counting: imaging and data analysis

For receptor counting, immunostained MET was imaged with the *d*STORM technique. For imaging a “switching buffer” containing oxygen scavenger (0.5 mg/mL glucose oxidase (Sigma), 40 μg/mL catalase (Sigma), 10% w/v glucose) and MEA (100 mM) in PBS was used. Super-resolution fluorescence microscopy was performed as described earlier [30] with the above mentioned experimental setup in TIR mode. Observation parameters were 10 000 frames recorded with 20 Hz using irradiation intensities of about 1 kW/cm^2^.

The number of InlB bound to receptor was analyzed using the background corrected photobleaching movies. Super-resolved images of the *d*STORM and photobleaching data were reconstructed using the rapi*d*STORM software. The resulting images were further analyzed in terms of particle numbers with the ImageJ plugin “3D object counter”.

## Competing interest

The authors declare that they have no conflict of interest.

## Authors’ contributions

HHN and MH designed research. MSD, DH, DMF and AG performed experiments. MSD and AG analyzed the data. MSD, HHN and MH wrote the manuscript. All authors read and approved the final manuscript.

## Supplementary Material

Additional file 1: Figure S1 *d*STORM imaging of MET receptor in fixed HeLa cells. MET was immunostained with Alexa Fluor 647. (**A**) Comparison of wide field and super-resolution *d*STORM images (scale bar 2 μm). (**B**) Enlarged section (2 × 2 μm²) of the inset in (**A**), (**C**) *d*STORM image of the section in (**B**). Click here for file

## References

[B1] Birchmeier C Birchmeier W Gherardi E Vande Woude GF Met, metastasis, motility and more Nat Rev Mol Cell Biol 2003 4 915 925 14685170 10.1038/nrm1261

[B2] Dramsi S Biswas I Maguin E Braun L Mastroeni P Cossart P Entry of Listeria monocytogenes into hepatocytes requires expression of InIB, a surface protein of the internalin multigene family Mol Microbiol 1995 16 251 261 7565087 10.1111/j.1365-2958.1995.tb02297.x

[B3] Shen Y Naujokas M Park M Ireton K InlB-Dependent Internalization of *Listeria* Is Mediated by the Met Receptor Tyrosine Kinase Cell 2000 103 501 510 11081636 10.1016/s0092-8674(00)00141-0

[B4] Braun L Dramsi S Dehoux P Bierne H Lindahl G Cossart P InlB: an invasion protein of Listeria monocytogenes with a novel type of surface association Mol Microbiol 1997 25 285 294 9282740 10.1046/j.1365-2958.1997.4621825.x

[B5] Niemann HH Jager V Butler PJ van den Heuvel J Schmidt S Ferraris D Gherardi E Heinz DW Structure of the human receptor tyrosine kinase Met in complex with the Listeria invasion protein InlB Cell 2007 130 235 246 17662939 10.1016/j.cell.2007.05.037

[B6] Schlessinger J Cell signaling by receptor tyrosine kinases Cell 2000 103 211 225 11057895 10.1016/s0092-8674(00)00114-8

[B7] Niemann HH Structural insights into Met receptor activation Eur J Cell Biol 2011 90 972 981 21242015 10.1016/j.ejcb.2010.11.014

[B8] Stamos J Lazarus RA Yao X Kirchhofer D Wiesmann C Crystal structure of the HGF beta-chain in complex with the Sema domain of the Met receptor EMBO J 2004 23 2325 2335 15167892 10.1038/sj.emboj.7600243 PMC423285

[B9] Gherardi E Sandin S Petoukhov MV Finch J Youles ME Ofverstedt LG Miguel RN Blundell TL Vande Woude GF Skoglund U Svergun DI Structural basis of hepatocyte growth factor/scatter factor and MET signalling Proc Natl Acad Sci U S A 2006 103 4046 4051 16537482 10.1073/pnas.0509040103 PMC1449643

[B10] Kong-Beltran M Stamos J Wickramasinghe D The Sema domain of Met is necessary for receptor dimerization and activation Cancer Cell 2004 6 75 84 15261143 10.1016/j.ccr.2004.06.013

[B11] Faletto DL Tsarfaty I Kmiecik TE Gonzatti M Suzuki T Woude GFV Evidence for noncovalent clusters of the c-Met protooncogene product Oncogene 1992 7 1149 1157 1317541

[B12] Banerjee M Copp J Vuga D Marino M Chapman T Van Der Geer P Ghosh P GW domains of the Listeria monocytogenes invasion protein InlB are required for potentiation of Met activation Mol Microbiol 2004 52 257 271 15049825 10.1111/j.1365-2958.2003.03968.x

[B13] Ferraris DM Gherardi E Di Y Heinz DW Niemann HH Ligand-mediated dimerization of the Met receptor tyrosine kinase by the bacterial invasion protein InlB J Mol Biol 2010 395 522 532 19900460 10.1016/j.jmb.2009.10.074

[B14] Niemann HH Petoukhov MV Hartlein M Moulin M Gherardi E Timmins P Heinz DW Svergun DI X-ray and neutron small-angle scattering analysis of the complex formed by the Met receptor and the Listeria monocytogenes invasion protein InlB J Mol Biol 2008 377 489 500 18262542 10.1016/j.jmb.2008.01.027

[B15] Hohlbein J Gryte K Heilemann M Kapanidis AN Surfing on a new wave of single-molecule fluorescence methods Phys Biol 2010 7 031001 20686191 10.1088/1478-3975/7/3/031001

[B16] Meckel T Semrau S Schaaf MJ Schmidt T Robust assessment of protein complex formation in vivo via single-molecule intensity distributions of autofluorescent proteins J Biomed Opt 2011 16 076016 21806277 10.1117/1.3600002

[B17] Ulbrich MH Isacoff EY Subunit counting in membrane-bound proteins Nat Methods 2007 4 319 321 17369835 10.1038/NMETH1024 PMC2744285

[B18] Ji W Xu P Li Z Lu J Liu L Zhan Y Chen Y Hille B Xu T Chen L Functional stoichiometry of the unitary calcium-release-activated calcium channel Proc Natl Acad Sci U S A 2008 105 13668 13673 18757751 10.1073/pnas.0806499105 PMC2533247

[B19] Zhang W Jiang Y Wang Q Ma X Xiao Z Zuo W Fang X Chen YG Single-molecule imaging reveals transforming growth factor-beta-induced type II receptor dimerization Proc Natl Acad Sci U S A 2009 106 15679 15683 19720988 10.1073/pnas.0908279106 PMC2747179

[B20] Teramura Y Ichinose J Takagi H Nishida K Yanagida T Sako Y Single-molecule analysis of epidermal growth factor binding on the surface of living cells EMBO J 2006 25 4215 4222 16946702 10.1038/sj.emboj.7601308 PMC1570442

[B21] Moshitch-Moshkovitz S Tsarfaty G Kaufman DW Stein GY Shichrur K Solomon E Sigler RH Resau JH Tsarfaty I Vande Woude GY In vivo direct molecular imaging of early tumorigenesis and malignant progression induced by transgenic expression of GFP-Met Neoplasia 2006 8 353 363 16790084 10.1593/neo.05634 PMC1592452

[B22] Pozner-Moulis S Pappas DJ Rimm DL Met, the hepatocyte growth factor receptor, localizes to the nucleus in cells at low density Cancer Res 2006 66 7976 7982 16912172 10.1158/0008-5472.CAN-05-4335

[B23] Michieli P Mazzone M Basilico C Cavassa S Sottile A Naldini L Comoglio PM Targeting the tumor and its microenvironment by a dual-function decoy Met receptor Cancer Cell 2004 6 61 73 15261142 10.1016/j.ccr.2004.05.032

[B24] Wickramasinghe D Kong-Beltran M Met Activation and Receptor Dimerization in Cancer Cell Cycle 2005 4 683 685 15846105 10.4161/cc.4.5.1688

[B25] Zarnegar R DeFrances MC Oliver L Michalopoulos G Identification and partial characterization of receptor binding sites for HGF on rat hepatocytes Biochem Biophys Res Commun 1990 173 1179 1185 2148475 10.1016/s0006-291x(05)80910-6

[B26] Mizuno K Higuchi O Tajima H Yonemasu T Nakamura T Cell density-dependent regulation of hepatocyte growth factor receptor on adult rat hepatocytes in primary culture J Biochem 1993 114 96 102 8407884 10.1093/oxfordjournals.jbchem.a124147

[B27] Higuchi O Nakamura T Identification and change in the receptor for hepatocyte growth factor in rat liver after partial hepatectomy or induced hepatitis Biochem Biophys Res Commun 1991 176 599 607 1827259 10.1016/s0006-291x(05)80226-8

[B28] Komada M Miyazawa K Ishii T Kitamura N Characterization of hepatocyte-growth-factor receptors on Meth A cells Eur J Biochem 1992 204 857 864 1311683 10.1111/j.1432-1033.1992.tb16705.x

[B29] Matsumoto K Kataoka H Date K Nakamura T Cooperative Interaction between α- and β-Chains of Hepatocyte Growth Factor on c-Met Receptor Confers Ligand-induced Receptor Tyrosine Phosphorylation and Multiple Biological Responses J Biol Chem 1998 273 22913 22920 9722511 10.1074/jbc.273.36.22913

[B30] Heilemann M van de Linde S Schüttpelz M Kasper R Seefeldt B Mukherjee A Tinnefeld P Sauer M Subdiffraction-resolution fluorescence imaging with conventional fluorescent probes Angewandte Chemie 2008 47 6172 6176 18646237 10.1002/anie.200802376

[B31] Göhler A Buchner C Andre S Doose S Kaltner H Gabius HJ Analysis of homodimeric avian and human galectins by two methods based on fluorescence spectroscopy: Different structural alterations upon oxidation and ligand binding Biochimie 2012 94 2659 2655 10.1016/j.biochi.2012.08.001 22884463

[B32] Machner MP Frese S Schubert W-D Orian-Rousseau V Gherardi E Wehland J Niemann HH Heinz DW Aromatic amino acids at the surface of InlB are essential for host cell invasion by Listeria monocytogenes Mol Microbiol 2003 48 1525 1536 12791136 10.1046/j.1365-2958.2003.03532.x

[B33] Ebbes M Bleymüller WM Cernescu M Nolker R Brutschy B Niemann HH Fold and function of the InlB B-repeat J Biol Chem 2011 286 15496 15506 21345802 10.1074/jbc.M110.189951 PMC3083156

[B34] Niemann HH Gherardi E Bleymuller WM Heinz DW Engineered variants of InlB with an additional leucine-rich repeat discriminate between physiologically relevant and packing contacts in crystal structures of the InlB:MET complex Protein Sci 2012 21 1528 1539 22887347 10.1002/pro.2142 PMC3526994

[B35] Adam G Delbrück M Rich A, Davidson N Reduction of Dimensionality in Biological Diffusion Processes Structural Chemistry and Molecular Biology 1968 San Francisco: W. H. Freeman and Company

[B36] Ziemba BP Knight JD Falke JJ Assembly of membrane-bound protein complexes: detection and analysis by single molecule diffusion Biochemistry 2012 51 1638 1647 22263647 10.1021/bi201743a PMC3318961

[B37] Niemann HH Structural basis of MET receptor dimerization by the bacterial invasion protein InlB and the HGF/SF splice variant NK1 Biochim Biophys Acta 2012 j.bbapap.2012.10.012 10.1016/j.bbapap.2012.10.012 23123275

[B38] Tolbert WD Daugherty J Gao C Xie Q Miranti C Gherardi E Vande Woude G Xu HE A mechanistic basis for converting a receptor tyrosine kinase agonist to an antagonist Proc Natl Acad Sci U S A 2007 104 14592 14597 17804794 10.1073/pnas.0704290104 PMC1965485

[B39] Siebold C Jones EY Structural insights into semaphorins and their receptors Semin Cell Dev Biol 2013 24 139 145 23253452 10.1016/j.semcdb.2012.11.003

[B40] Merlin S Pietronave S Locarno D Valente G Follenzi A Prat M Deletion of the ectodomain unleashes the transforming, invasive, and tumorigenic potential of the MET oncogene Cancer Sci 2009 100 633 638 19175607 10.1111/j.1349-7006.2008.01079.x PMC11158143

[B41] Endres NF Engel K Das R Kovacs E Kuriyan J Regulation of the catalytic activity of the EGF receptor Curr Opin Struct Biol 2011 21 777 784 21868214 10.1016/j.sbi.2011.07.007 PMC3232302

[B42] Li E Hristova K Receptor tyrosine kinase transmembrane domains Cell Adh Migr 2010 4 249 254 20168077 10.4161/cam.4.2.10725 PMC2900622

[B43] Livnah O Crystallographic evidence for preformed dimers of erythropoietin receptor before ligand activation Science 1999 283 987 990 9974392 10.1126/science.283.5404.987

[B44] Brown RJ Adams JJ Pelekanos RA Wan Y McKinstry WJ Palethorpe K Seeber RM Monks TA Eidne KA Parker MW Waters MJ Model for growth hormone receptor activation based on subunit rotation within a receptor dimer Nat Struct Mol Biol 2005 12 814 821 16116438 10.1038/nsmb977

[B45] Pang X Zhou HX A common model for cytokine receptor activation: combined scissor-like rotation and self-rotation of receptor dimer induced by class I cytokine PLoS Comput Biol 2012 8 e1002427 22412367 10.1371/journal.pcbi.1002427 PMC3297564

[B46] Sako Y Minoguchi S Yanagida T Single-molecule imaging of EGFR signalling on the surface of living cells Nat Cell Biol 2000 2 168 172 10707088 10.1038/35004044

[B47] Chung I Akita R Vandlen R Toomre D Schlessinger J Mellman I Spatial control of EGF receptor activation by reversible dimerization on living cells Nature 2010 464 783 787 20208517 10.1038/nature08827

[B48] Gherardi E Youles ME Miguel RN Blundell TL Iamele L Gough J Bandyopadhyay A Hartmann G Butler PJ Functional map and domain structure of MET, the product of the c-met protooncogene and receptor for hepatocyte growth factor/scatter factor Proc Natl Acad Sci U S A 2003 100 12039 12044 14528000 10.1073/pnas.2034936100 PMC218709

[B49] Göhler A Andre S Kaltner H Sauer M Gabius HJ Doose S Hydrodynamic properties of human adhesion/growth-regulatory galectins studied by fluorescence correlation spectroscopy Biophys J 2010 98 3044 3053 20550917 10.1016/j.bpj.2010.03.040 PMC2884264

[B50] de Chaumont F Dallongeville S Chenouard N Herve N Pop S Provoost T Meas-Yedid V Pankajakshan P Lecomte T Le Montagner Y Icy: an open bioimage informatics platform for extended reproducible research Nat Methods 2012 9 690 696 22743774 10.1038/nmeth.2075

