# Supplementary material for: Single-molecule photobleaching reveals increased MET receptor dimerization upon ligand binding in intact cells
Source: BMC Biophys. 2013 Jun 3;6:6. doi: 10.1186/2046-1682-6-6 (PMC3674922; doi:10.1186/2046-1682-6-6)
Supplement: Additional file 1: Figure S1 — dSTORM imaging of MET receptor in fixed HeLa cells. MET was immunostained with Alexa Fluor 647. (A) Comparison of wide field and super-resolution dSTORM images (scale bar 2 μm). (B) Enlarged section (2 × 2 μm²) of the inset in (A), (C) dSTORM image of the section in (B). [file 2046-1682-6-6-S1.docx]

Title: **Single-molecule photobleaching reveals MET receptor dimerization after ligand binding on intact cells**

Running title: Single-molecule photobleaching of MET dimers

Authors: Marina S. Dietz^1^, Daniel Haße^2^, Davide M. Ferraris^3^, Antonia Göhler^4^, Hartmut H. Niemann^2^, and Mike Heilemann^1^

Addresses: ^1^Institute of Physical and Theoretical Chemistry, Johann Wolfgang Goethe-University, Max-von-Laue-Str. 7, 60438 Frankfurt, Germany

^2^Structural Biochemistry, Department of Chemistry, Bielefeld University, Universitaetsstrasse 25, 33615 Bielefeld, Germany

^3^Department of Pharmaceutical Science, University of Eastern Piedmont “Amedeo Avogadro”, Via Bovio 6, 28100 Novara, Italy

^4^Department of Biotechnology and Biophysics, Julius-Maximilians University, Am Hubland, Biozentrum, 97074 Wuerzburg, Germany

Correspondence: Mike Heilemann, Hartmut Niemann

Telephone: +49 69 798 29736 (MH); +49 521 106 6154 (HN)

Fax: +49 69 798 29560 (MH); +49 521 106 6146 (HN)

E Mail: [heilemann@chemie.uni-frankfurt.de](mailto:heilemann@chemie.uni-frankfurt.de) ; [hartmut.niemann@uni-bielefeld.de](mailto:hartmut.niemann@uni-bielefeld.de)

**SUPPLEMENTARY INFORMATION**

**SUPPLEMENTAL FIGURES**

***Figure S1***

***
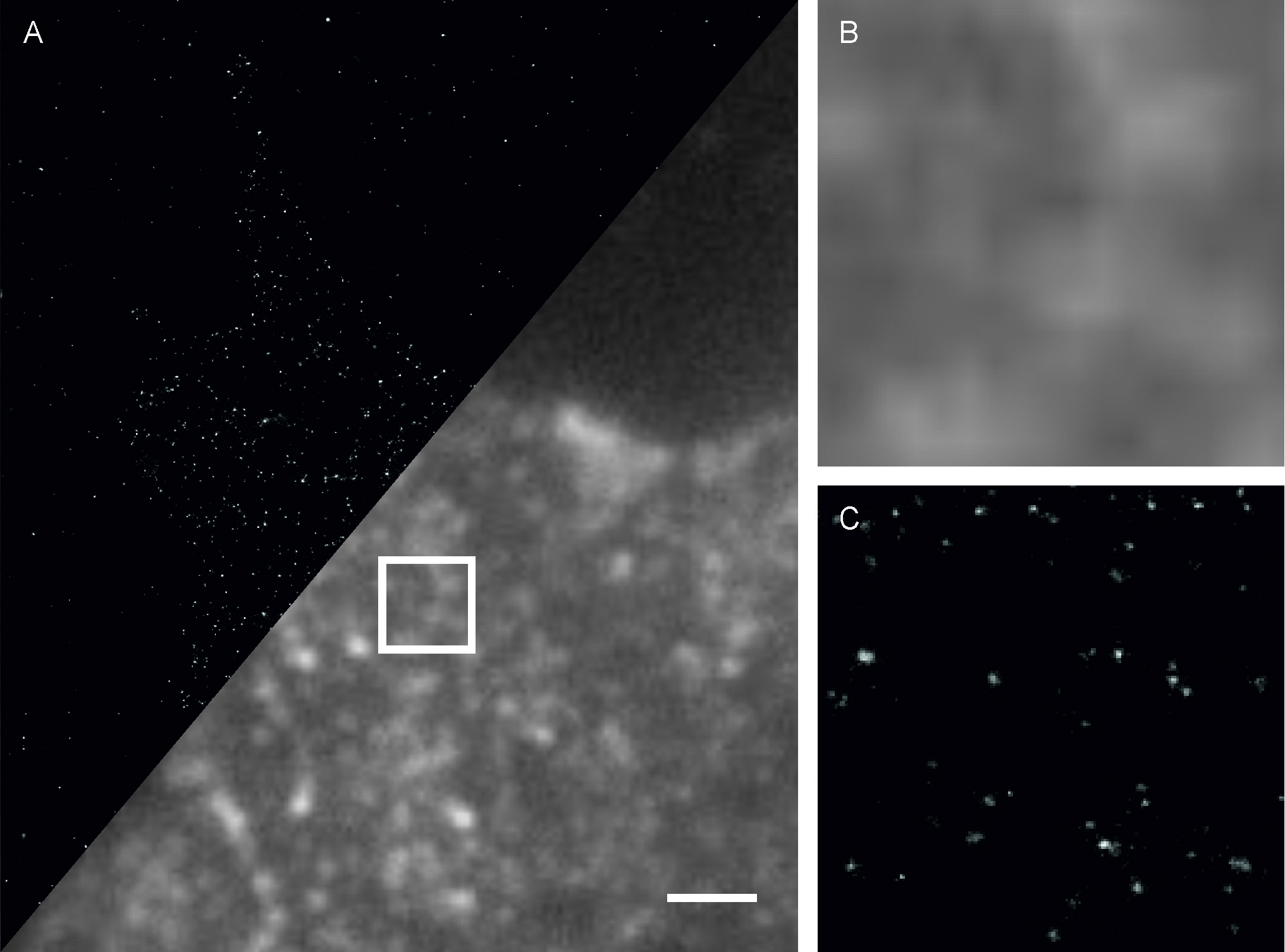
***

***d*STORM imaging of MET receptor in fixed HeLa cells.**

MET was immunostained with Alexa Fluor 647. (A) Comparison of wide field and super-resolution *d*STORM images (scale bar 2 µm). (B) Enlarged section (2 x 2 µm²) of the inset in (A), (C) *d*STORM image of the section in (B).
